# The Effect of A-Cation and X-Anion Substitutions on the Electronic and Structural Properties of A_2_ZrX_6_ ‘Defect’ Perovskite Materials: A Theoretical Density Functional Theory Study

**DOI:** 10.3390/ma18030726

**Published:** 2025-02-06

**Authors:** Christina Kolokytha, Nektarios N. Lathiotakis, Andreas Kaltzoglou, Ioannis D. Petsalakis, Demeter Tzeli

**Affiliations:** 1 Theoretical and Physical Chemistry Institute, National Hellenic Research Foundation, 48 Vassileos Constantinou Ave., GR-11635 Athens, Greece; kolokythac28@chem.uoa.gr (C.K.); akaltzoglou@eie.gr (A.K.); idpet@eie.gr (I.D.P.); 2 Laboratory of Physical Chemistry, Department of Chemistry, National and Kapodistrian University of Athens, GR-15784 Zografou, Greece

**Keywords:** hybrid organic-inorganic materials, optoelectronic properties, hybrid DFT calculations

## Abstract

In the present work, nine ‘defect’ perovskites with the chemical formula A_2_ZrX_6_ have been studied, where the A-site cations are a methylammonium cation, formamidinium cation, and trimethyl-sulfonium cation and the X-site anions are halogen, X = Cl, Br, and I. We employ periodic DFT calculations using GGA-PBE, MBJ, HSEsol, and HSE06 functionals. All studied compounds exhibit a wide-bandgap energy that ranges from 5.22 eV to 2.11 eV, while for some cases, geometry optimization led to significant structural modification. It was found that the increase in the halogen size resulted in a decrease in the bandgap energy. The choice of the organic A-site cation affects the bandgap as well, which is minimal for the methylammonium cation. Such semiconductors with organic cations may be utilized in optoelectronic devices, given the substantial benefit of solution processability and thin film formation compared to purely inorganic analogs, such as Cs_2_ZrX_6_.

## 1. Introduction

In recent decades, there has been substantial interest in perovskite materials, encompassing both experimental and computational studies [1,2,3,4]. Numerous recent studies have focused on developing new materials and examining the crystal structure and optoelectronic and thermoelectric properties of perovskites. These materials have garnered attention due to their promising features, including low-cost fabrication, adjustable bandgap energies, high absorption coefficients, impressive defect tolerance, and their lightweight, flexible nature, making them ideal for applications where weight and form factor are crucial. The most common applications of perovskites are in laser crystals [5], light-emitting diodes (LEDs) [5,6], solar cells [7], transistors [8], memories [9], superconductors [10], photocatalysts [11], photo electrolysis [4], photodetectors (PDs), and other optoelectronic devices [4,12].

Metal-halide perovskites with the chemical formula ABX_3_, where the A-site contains an inorganic cation such as cesium or an organic one such as the methylammonium cation (MA) or the formamidinium cation (FA), the B-site typically contains a divalent metal like Pb^2+^, Sn^2+^, or Ge^2+^, while the X site represents a halogen anion (Cl, Br, or I), have been considered very promising in solar energy conversion for third-generation solar cells [8]. In these systems, the A-site cations appear in twelve-fold coordination with the anions, while the B-site cations are in six-fold coordination. The power conversion efficiency of perovskite solar cells has seen a notable rise, reaching up to 20% in some cases [13,14,15]. Apart from solar cells, metal-halide perovskites are also well-suited for use in light-emitting devices: In 2015, Stranks and Snaith explored three-dimensional organic–inorganic metal-halide perovskites (MAPbI_3−x_Cl_x_ and MAPbI_3−x_Br_x_), which exhibited internal quantum efficiencies ranging from 0.4% to 3.5% and emitted light in the near-infrared and green spectra [7]. Serious challenges to be addressed for metal-halide perovskites are the metal toxicity (e.g., in the case of Pb) and their stability. Aiming to address the stability of such compounds, the use of trimethylsulfonium ((CH_3_)_3_S) cation (TMS) as a substitute for the hygroscopic cations has been proposed [16], leading to a series of (CH_3_)_3_SPbX_3_ compounds that are very stable at ambient conditions but with a bandgap that exceeds 3 eV, limiting their performance as absorbers in solar cells.

Another subfamily of perovskites (known also as ‘defect’ perovskites of the K_2_PtCl_6_ archetype) with the stoichiometry A_2_BX_6_, where B is a tetravalent metal cation (Zr, Sn, or Te), has also received significant attention. These compounds hold significant potential for a broad range of applications [12,17]. Cucco et al. theoretically investigated the electronic properties of Cs_2_BX_6_, where B = Sn, Te, Zr, and found tunable electronic and optical properties for hole and electron transport [12]. Abfalterer et al. theoretically synthesized and studied Cs_2_ZrX_6_ (X = Cl, Br) perovskite derivatives, observing a very good agreement between theory and experiment [17]. The obtained bandgaps for these systems were found to be indirect, with values of 5.06 and 3.91 eV for Cl and Br, respectively. Dai et al. reported observing the novel metal halide ((CH_3_)_4_N)_2_ZrCl_6_ exhibiting excitation-dependent luminescence across the full visible region [18]. Lin et al. explored the optical regulation of A_2_ZrX_6_ perovskites, focusing on the host–guest interaction effect by introducing several guestdoping ions, providing insights into the compositional engineering of such compounds [19]. Recently, Tagiara et al. synthesized yet another member of this subfamily, (TMS)_2_ZrCl_6,_ with broad photoluminescence [20]. Zr-based compounds are yet largely unexplored, in contrast to their Sn (IV) analogs. Most research so far has been performed on Cs_2_ZrCl_6_, some has been performed on Cs_2_ZrBr_6,_ and very limited has been focused on Cs_2_ZrI_6_; mainly, ZrI_4_ and its adducts are not stable in the air and need to be handled strictly under inert conditions. In contrast to the Cs_2_ZrX_6_ that are prepared by melting the inorganic precursors at ca. 500 °C [21,22], the synthesis of their organic counterparts A_2_ZrX_6_ (A = TMS, MA, and FA) can only be performed at lower temperatures up to ca. 150 °C, either in solid state or in organic solutions. However, the recent synthesis of TMS_2_ZrCl_6_ [20] opens the way for further experimental exploitation in this class of compounds.

Perovskite materials are categorized according to their bandgap energy into three groups: (a) narrow-bandgap perovskites (E_gap_ < 1.4 eV), (b) intermediate bandgap perovskites (E_gap_ around 1.4–1.6 eV), and (c) wide-bandgap (WBG) perovskites (E_gap_ > 1.7 eV). The bandgap is crucial for the determination of the material applications. Thus, materials with a bandgap of about 4–5 eV are considered wide-bandgap semiconductors, with applications as UV detectors and high temperature sensors, in the design of deep UV light-emitting diodes, and in high-power electronics, optoelectronics, etc. Materials with a bandgap around 3.5 eV are also considered appropriate as photocatalysts, UV detectors, UV lasers, thermal sensors, in optoelectronic devices, and in deep UV light-emitting diodes. Materials with a bandgap around 2.8–1.5 eV present many applications in solar cells, as photocatalysts, in LEDs, in optoelectronics, in photovoltaics for blue and UV, and as thin-film transistors. Specifically, a bandgap around 2.5 eV allows it to absorb visible light in the blue, a bandgap of 1.9 eV allows it to absorb visible light, particularly toward the red, while a bandgap of 1.5 eV is particularly well suited for a range of energy and optoelectronic applications due to its ability to efficiently absorb and emit visible and near-infrared light [23,24].

In the present computational study, we investigate metal-halide perovskites with the chemical formula A_2_ZrX_6_, where the A-site cations are the methylammonium cation (MA^+^; CH_3_NH_3_^+^), the formamidinium cation (FA^+^; CH(NH_2_)_2_^+^), and the trimethylsulfonium cation (TMS^+^; (CH_3_)_3_S^+^) and the X are anions of halogens. Our aim is to reveal the effect of substituting the halogen anion (X-site anion), i.e., Cl^-^, Br^-^, and I^-^, as well as the A-site cation on the calculated bandgap.

## 2. Computational Details

The present theoretical study is based on DFT calculations using the Viena Ab initio Simulation Package (VASP) code, which implements the projected augmented wave (PAW) pseudopotential method [25,26,27]. Every structure was energetically optimized at the generalized gradient approximation (GGA) level, employing the Perdew–Burke–Ernzerhof (PBE) exchange-correlation functional [28]. Based on the agreement of the structural properties obtained with the PBE functional to the experimental ones for TMS_2_ZrCl_6_ and to avoid computational complexity and convergence issues one might encounter with hybrid functionals, all structures in this work were optimized using PBE approximation. For the electronic properties (band structure and DOS), due to the systematic and well-documented inaccuracy of the GGA level of theory, we employed the modified Becke–Johnson approximation (mBJ) [29,30] and screened hybrid functionals like Heyd–Scuseria–Ernzerhof HSE06 [31] and HSEsol [32]. We used the value of 400 eV for the maximum energy cutoff and a 2 × 2 × 2 reciprocal space sampling. Moreover, our results concerning both the optimal structures and the bandgaps are highly converged with respect to reciprocal space sampling and energy cutoff. As we found, after changing the cutoff from 400 to 600 eV or the k-sampling from 2 × 2 × 2 to 4 × 4 × 4, the lattice constant variation is of the order of 1% while the bandgap is essentially unaffected.

## 3. Results and Discussion

### 3.1. TMS_2_ZrX_6_ (X = Cl, Br, I)

**Figure 1:**
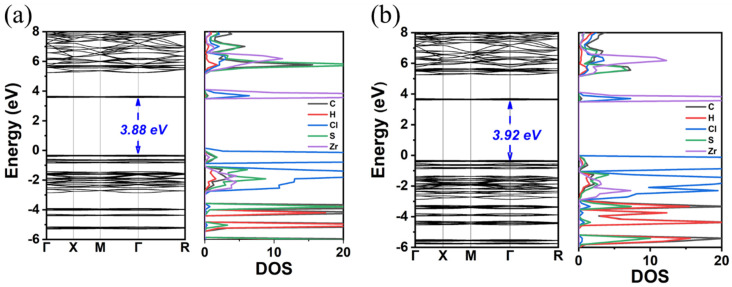
Calculated band structure and DOS of the (**a**) experimental crystal structure TMS_2_ZrCl_6_ and (**b**) energetically optimized crystal structure TMS_2_ZrCl_6_.

At first, the experimentally determined crystal structure of TMS_2_ZrCl_6_ (data in SI of Ref. [20] as initial model) was fully energetically optimized using the GGA-PBE. The calculated structural characteristics, i.e., crystal symmetry, lattice parameters, and atomic coordinates, are in good agreement with the experimental ones [20]. For instance, the structural optimization resulted in a small reduction in the cell dimension by 0.028 Å compared to the experimental value. As expected, the calculated bandgap energy (E_gap_) with GGA-PBE is not in agreement with the experimental one. Specifically, with GGA-PBE, we found a bandgap value of 3.88 eV for the experimental crystal structure and 3.92 eV for the optimized structure (see Figure 1). These bandgaps were found to be direct, and they are significantly smaller than the experimental value of 5.1 eV [20]. Note that the small increase in the bandgap energy is consistent with the fact that the optimization of the structure does not significantly affect the structural properties of the material.

**Figure 2:**
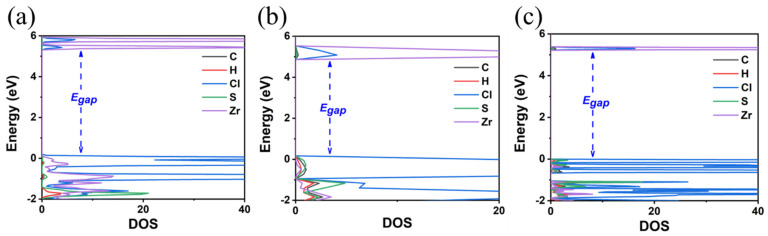
Calculated DOS of TMS_2_ZrCl_6_ using three different functionals: (**a**) Meta-GGA functional mBJ, (**b**) screened hybrid functional HSEsol, and (**c**) screened hybrid functional HSE06.

As already mentioned, we calculated the electronic properties using mBJ, HSE06, and HSEsol approximations and adopting the structure that was optimized by the GGA-PBE functional. Our results are shown in Figure 2 and Table 1. As we see, these approximations significantly improve the values of E_gap_. These values obtained by mBJ and HSE06 are 5.23 and 5.22 eV, respectively, approaching the experimental value of 5.1 eV. Note that attempts to optimize the structure via the mBJ, HSEsol, and HSE06 functionals either failed to converge or were particularly time-consuming. Thus, we keep our single-point calculations using the GGA-PBE-optimized structure, which was very similar to the experimental one.

**Figure 3:**
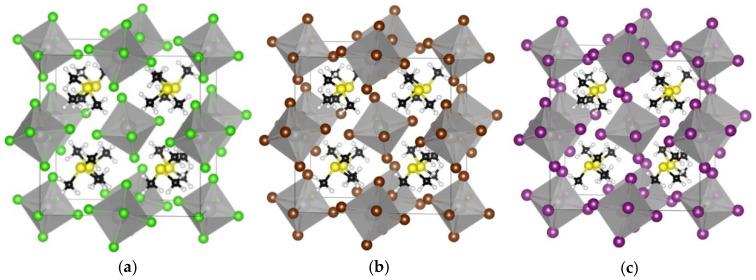
The optimized cubic crystal structures are shown for (**a**) TMS_2_ZrCl_6_, (**b**) TMS_2_ZrBr_6_, and (**c**) TMS_2_ZrI_6_. The images were created using VESTA software, version 3.90.1a [35]. Color assignment: yellow for S, black for carbon, white for hydrogen, grey for Zr, green for Cl, brown for Br, and violet for I.

Furthermore, we studied the effect of X-site anion replacement on the electronic and structural properties of the TMS_2_ZrX_6_. The three different crystal structures of TMS_2_ZrCl_6_, TMS_2_ZrBr_6,_ and TMS_2_ZrI_6_ are plotted in Figure 3. The corresponding lattice constants were calculated at 12.65 Å, 12.86 Å, and 13.49 Å, showing an increase of about 0.2 Å, due to the replacement of the Cl with the Br, and an additional increase of 0.6 Å, due to the replacement with the I anion. In all three compounds, the [ZrX_6_]^2-^ anions deviate only slightly from regular octahedral symmetry, with equatorial X–Zr–X angles at ca. 89.65° and 90.35°. The TMS cations retain their C3 rotational axis and adopt certain orientations in the unit cell (through this C3 axis and the electron lone pair of the S atom) with regard to the [111] direction. The octahedra tilt significantly, namely by ca. 30°, considering the Zr–X–Zr angle for two octahedra along the [100] direction compared to the K_2_PtCl_6_ archetype. This tilt is stereochemically induced by the large TMS cation, without altering, though, the coordination environment of the Zr cations [33,34].

**Figure 4:**
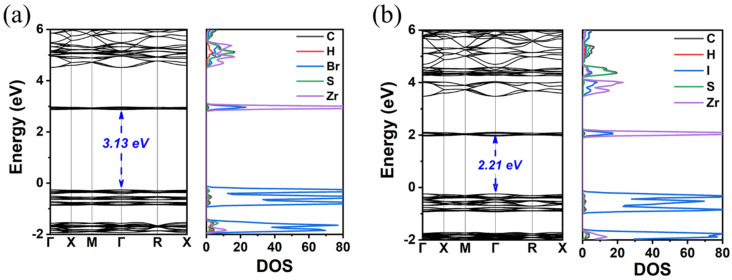
The calculated GGA-PBE band structure and DOS of (**a**) TMS_2_ZrBr_6_ and (**b**) TMS_2_ZrI_6_.

**Figure 5:**
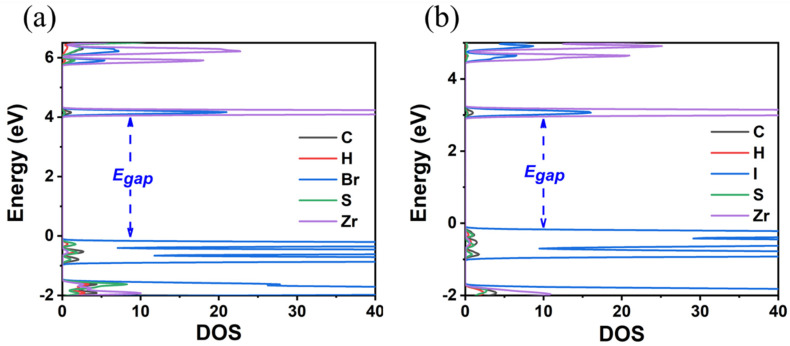
The calculated HSE06 DOS of (**a**) TMS_2_ZrBr_6_ and (**b**) TMS_2_ZrI_6_.

Finally, the replacement of the X-site anion from Cl to Br and finally to I results in a decrease in the E_gap_. Specifically, it was found that the reduction is Cl to Br 0.8(1.2) eV and Br to I 0.9(1.1) eV using the GGA-PBE(HSE06) functionals; see Figure 4 and Figure 5 and Table 2. Given that for TMS_2_ZrCl_6,_ the HSE06 E_gap_ was calculated at 5.22 eV, in excellent agreement with the experimental gap of 5.1 eV, this functional is our best choice for the calculation of the bandgap energies, and we conclude that the E_gap_ value of the TMS_2_ZrBr_6_ and TMS_2_ZrI_6_ are 4.05 eV and 2.97 eV, respectively; see Table 2. For comparison, the isostructural Sn compounds TMS_2_SnX_6_ exhibit experimental bandgaps of 4.1, 2.9, and 1.4 eV for X = Cl, Br, and I, respectively, and theoretical bandgaps of ca. 3.0, 2.0, and 0.9 eV for X = Cl, Br, and I, respectively, [36]. This effect is attributed mainly to more diffuse Sn orbitals compared to the Zr orbitals.

To sum up, we conclude that the increase in the atomic radius of the halogens (Cl < Br < I) results in an increase in the size of the unit cell by about 0.2–0.5 Å in each case. The halogen–halogen distance in the same octahedra increases when the atomic weight of the halogen increases. The decrease in the halogen electronegativity (Cl < Br < I) results in a decrease in the bandgap energy by about 1 eV in each case. Our best bandgap energies are obtained via the HSE06 functional, while the GGA-PBE significantly underestimated the E_gap_ values.

### 3.2. MA_2_ZrX_6_ (X = Cl, Br, I)

**Figure 6:**
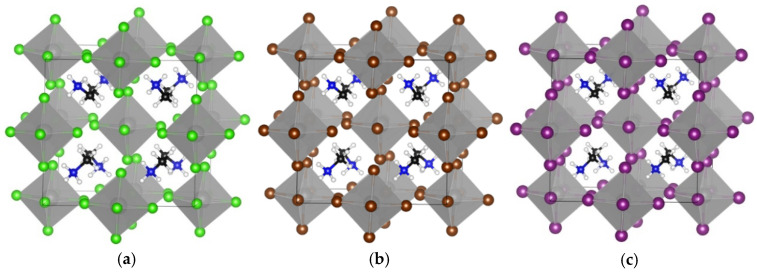
The optimized crystal structures of (**a**) MA_2_ZrCl_6_, (**b**) MA_2_ZrBr_6_, and (**c**) MA_2_ZrI_6_. The images were created using VESTA software, version 3.90.1a. Color assignment: blue for N, black for carbon, white for hydrogen, grey for Zr, green for Cl, brown for Br, and violet for I.

Additionally, we studied how the A-site cation replacement affects the structural and optoelectronic properties of A_2_ZrX_6_. In particular, the crystal structures of MA_2_ZrX_6_ were studied with three different halogens in the X-site anion, i.e., X = Cl, Br, and I. The obtained optimized crystal structures are all triclinic, see Figure 6 and Table 3, contrary to those of TMS_2_ZrX_6_, which are all cubic, as we have seen (Figure 3 and Table 2). This is attributed to the low symmetry of the MA cation and to the fact that as the structural refinement results in a certain orientation in order to form H bonds with the halogen atoms of the inorganic framework, the unit cell drops to triclinic symmetry. It is of note that the actual crystal structures of most perovskite compounds that contain MA and FA cations exhibit crystallographic disorder at room temperature due to the rotation of the cations [37]. The values of the lattice constants of the MA_2_ZrX_6_, see Table 3, increase with the replacement of the Cl with Br and finally with the I anion; similarly to the case of TMS_2_ZrX_6_, the lattice constants present an increase of about 0.4 Å, due to the replacement of Cl with Br, and an additional increase of 0.7 Å, due to the replacement of Br with I. The inorganic octahedra almost retain their regular shape, except for very small deviations in terms of both the angles and bond lengths. A minor tilt of the octahedra of less than 1° is observed by using the same Zr–X–Zr notation as described above for TMS_2_ZrX_6_.

**Figure 7:**
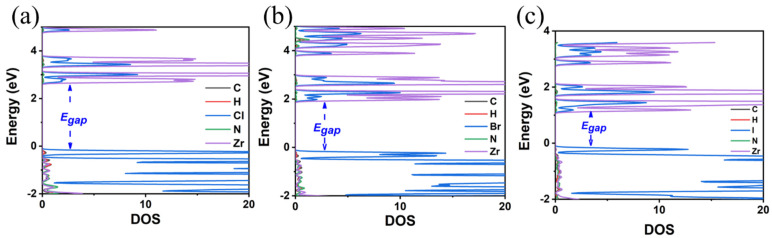
The calculated DOS of (**a**) MA_2_ZrCl_6_, (**b**) MA_2_ZrBr_6_, and (**c**) MA_2_ZrI_6_ using the GGA-PBE functional.

**Figure 8:**
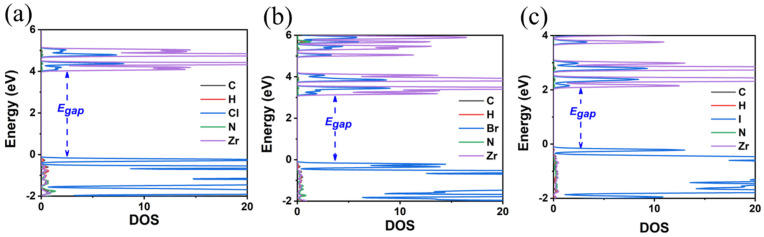
The calculated DOS of (**a**) MA_2_ZrCl_6_, (**b**) MA_2_ZrBr_6_, and (**c**) MA_2_ZrI_6_ using the HSE06 functional.

Regarding the E_gap_ values, as in the case of TMS_2_ZrX_6_, the replacement of the X-site anion from Cl to Br and finally to I results in a decrease in the E_gap_ by about 1 eV; see Figure 7 and Figure 8. The HSE06 functional is our best choice for calculating bandgap energies, and our predictions are 4.06 eV for MA_2_ZrCl_6_, 3.14 eV for MA_2_ZrBr_6_, and 2.11 eV for MA_2_ZrI_6_. Again, the GGA-PBE underestimates the bandgap energies by about 1 eV compared to HSE06, cf. Figure 7 and Figure 8. Again, all bandgaps are direct.

Comparing the MA_2_ZrX_6_ and the TMS_2_ZrX_6_ systems, it was found that the MA_2_ZrX_6_ presents significantly smaller bandgap energies by about 1 eV than the corresponding TMS_2_ZrX_6_ systems. This can be partly explained by the presence of a nitrogen atom in the MA, which is more electronegative than the sulfur atom found in the TMS, but also by the fact that the MA alters the crystal structure. Its smaller size compared to the TMS plays an important role in this structural alteration. Additionally, in both MA_2_ZrX_6_ and TMS_2_ZrX_6_, the primary contribution to VBM states comes from the halogen p-orbitals, while that of the CBM comes from the Zr d-orbitals. Although the contribution of the MA orbitals to the valence and conduction bands are small, the TMS to MA substitution substantially affects the structural features of the systems and, hence, indirectly influences the electronic properties, as indicated by the significant decrease in the bandgap energy.

### 3.3. FA_2_ZrX_6_ (X = Cl, Br, I)

**Figure 9:**
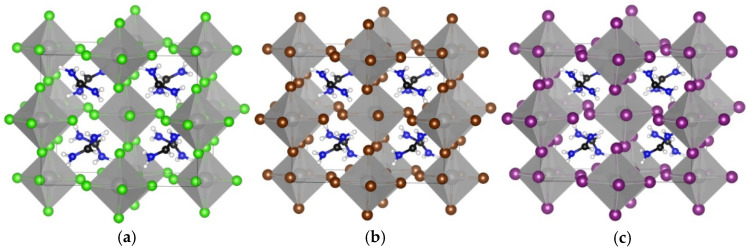
The optimized crystal structures are shown for (**a**) FA_2_ZrCl_6_, (**b**) FA_2_ZrBr_6_, and (**c**) FA_2_ZrI_6_. The images were created using VESTA software, version 3.90.1a. Color assignment: blue for N, black for carbon, white for hydrogen, grey for Zr, green for Cl, brown for Br and violet for I.

Finally, the crystal structures of A_2_ZrX_6_ were calculated, revealing the formamidinium cation in the A-site and X = Cl, Br, and I (Figure 9). The calculated optimal structures are all triclinic, as in the case of the MA, which is also due to the lower symmetry of the FA compared to the TMS. The lattice parameters and structural characteristics are given in Table 4. A tilt of ca. 5° from the ideal angle of 180° is observed in the octahedra. The FA groups are oriented diagonally between the adjacent inorganic layers, and the C atoms sit almost in the center of the cuboctahedral voids, which is the most symmetric and apparently thermodynamically favorable position.

**Figure 10:**
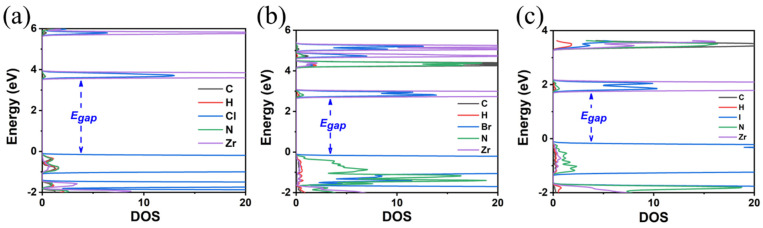
The calculated DOS of (**a**) FA_2_ZrCl_6_, (**b**) FA_2_ZrBr_6_, and (**c**) FA_2_ZrI_6_ using the GA-PBE functional.

**Figure 11:**
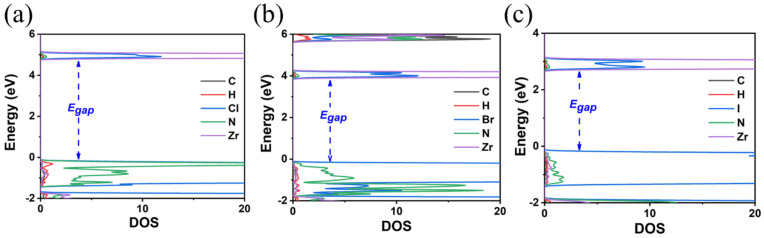
The calculated DOS of (**a**) FA_2_ZrCl_6_, (**b**) FA_2_ZrBr_6_, and (**c**) FA_2_ZrI_6_ using the HSE06 functional.

In the final A-site cation substitution, both the electronic and structural properties are affected. The formamidinium cation, containing two electronegative nitrogen atoms, influences the bandgap energy. The E_gap_ values are decreased compared to the TMS as the size of the halogen is increased, but are larger than those for the MA, as seen in Figure 10 and Figure 11 and Table 4. DOS calculations using the HSE06 functional (see Figure 11) show that, for the FA, there is a significant contribution from N p-orbitals to the states close to VMB. This contribution is more pronounced for the Cl anion and decreases for Br and even more so for I. The HSE06 functional predicts bandgap energies of 4.81 eV (FA_2_ZrCl_6_), 3.89 eV (FA_2_ZrBr_6_), and 2.54 eV (FA_2_ZrI_6_).

## 4. Trends

Two key findings emerge from this study. The first one concerns the effect of A-site cation substitution, and the second one concerns the effect of halogen substitution on the structural and electronic properties of the compounds.

*A-site cation substitution*: Regarding the structural characteristics, the A_2_ZrX_6_ structure type exhibits high stereochemical flexibility in order to accommodate different size cations, namely, in order of increasing sizes, Cs < MA < FA < TMS. Apart from the unit cell expansion and the change from the cubic to triclinic unit cell when incorporating cations with no symmetry (e.g., MA and FA), the octahedra tilt in order to accommodate large cations, such as the TMS. An alternative approach with regard to the volume of the organic cations, that takes into account the dynamic disorder within the crystal structure, is introduced by the term ‘effective ionic radius’ [38]. Based on this, the MA has an effective ionic radius of 2.17 Å and an FA of 2.53 Å. This difference also explains the larger unit cell volume found for the FA_2_ZrX_6_ series compared to the MA_2_ZrX_6_ series.

**Figure 12:**
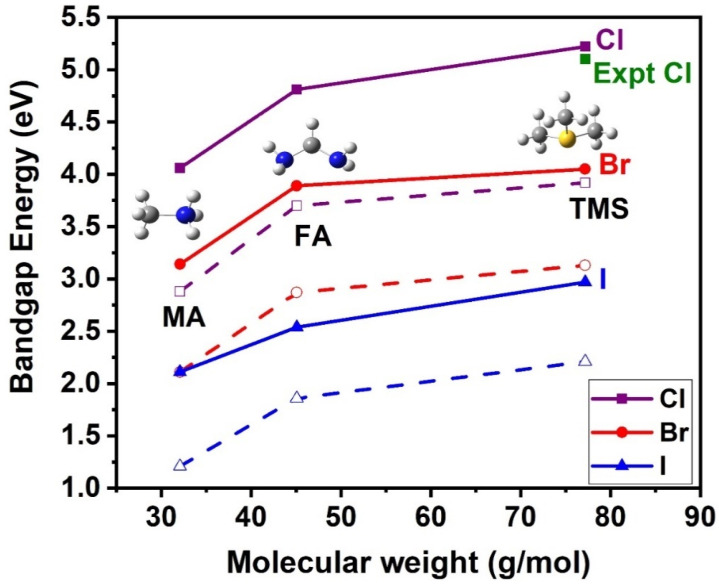
Bandgap energy as a function of the molecular weight of the A-site cation (g/mol) for the A_2_ZrX_6_ crystal, calculated using the HSE06 (solid lines) and GGA-PBE (dash lines) functionals. Available experimental value is also included [20]. Color assignment: blue for N, grey for carbon, white for hydrogen, yellow for S.

Changing the type and the effective ionic radius of the A-site cation substantially affects the bandgap energy, as seen in Figure 12. Note that both GGA-PBE and HSE06 present the same trends, although GGA-PBE significantly underestimates the gaps. In the case of the TMS^+^, which is the biggest A-site cation and includes a sulfur atom, the bandgap is the largest. In the cases of the MA and FA, with one or two nitrogen atoms (more electronegative atoms), the bandgap decreases. The bandgaps for the MA are the smallest, indicating that E_gap_ correlates also with the size of the A-site cation, decreasing as the size decreases.

**Figure 13:**
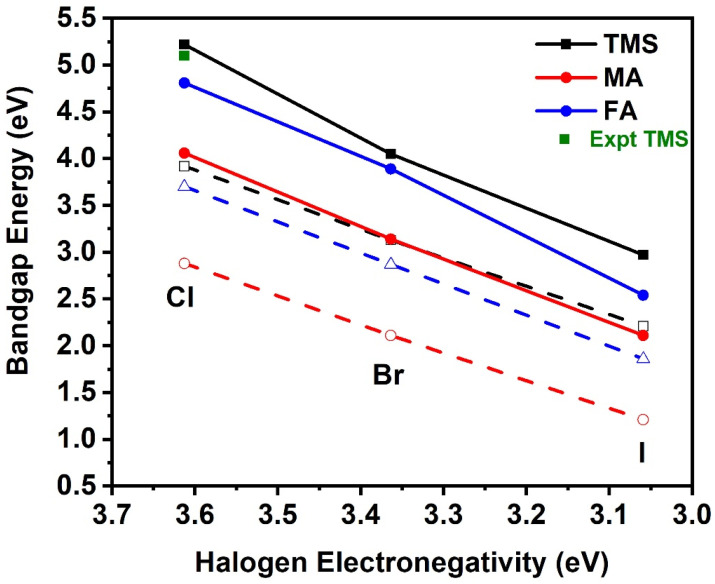
Bandgap energy as a function of halogen electronegativity of the A_2_ZrX_6_, calculated using the HSE06 (solid lines) and GGA-PBE (dash lines) functionals. Available experimental value is also included [20].

*X-site anion substitution:* The substitution of the X-site anion affects the electronic properties of the crystal structures. As the electronegativity of the X-site halogen is decreased, i.e., Cl > Br > I, the bandgap also decreases; see Figure 13. Both GGA-PBE and HSE06 present similar orbital type contributions for the VBM and CBM states. Our best results are obtained with the HSE06, which, in the case of the TMS_2_ZrX_6_ crystal, where experimental E_gap_ is available, the HSE06 E_gap_ is in excellent agreement with the experimental gap.

All the studied structures exhibit a wide range of bandgap energy that ranges from 5.22 eV to 2.11 eV. These perovskites are wide-bandgap semiconductors and, depending on their bandgap energy, are suitable for many applications in optoelectronics.

## 5. Conclusions

In the present paper, nine perovskites with the chemical formula A_2_ZrX_6_ have been studied, where the A-site cations are the methylammonium cation (MA^+^), formamidinium cation (FA^+^), and trimethylsulfonium cation (TMS^+^) and the X-site anions are halogen, X = Cl, Br, and I. The crystal structures were studied using periodic DFT calculations with GGA-PBE, MBJ, HSEsol, and HSE06 functionals, employing the projected augmented wave (PAW) pseudopotential method. Our aim was to study a series of materials to determine the influence of the A-site cation or the X-site anion substitution on the bandgap energy and identify materials suitable for specific applications.

Our best bandgap energies were obtained with the HSE06 functional, which is in excellent agreement with the experiment concerning the bandgap energy of the TMS_2_ZrCl_6_ structure, while the GGA-PBE significantly underestimated the E_gap_ by about 1 eV. Our predicted bandgap energies of the studied systems are 5.22 eV (TMS_2_ZrCl_6_; expt: 5.1 eV [20]), 4.05 eV (TMS_2_ZrBr_6_), 2.97 eV (TMS_2_ZrI_6_), 4.06 eV (MA_2_ZrCl_6_), 3.14 eV (MA_2_ZrBr_6_), 2.11 eV (MA_2_ZrI_6_), 4.81 eV (FA_2_ZrCl_6_), 3.89 eV (FA_2_ZrBr_6_), and 2.54 eV (FA_2_ZrI_6_). For comparison, the experimentally and theoretically studied Cs_2_ZrX_6_ compounds show bandgap values in the range of ca. 5 eV for Cs_2_ZrCl_6_ [17], ca. 3.8 eV for Cs_2_ZrBr_6_ [17], and ca. 3–3.7 eV for Cs_2_ZrBr_6_ [12].

Regarding the effect of halogen substitution on the structural and electronic properties of the considered systems, it was found that: The decrease in the halogen electronegativity (Cl < Br < I) or the increase in the halogen size results in an expansion of the unit cell and a decrease in the bandgap energy by about 1 eV in each case. Our result is consistent with the known trend of bandgap lowering in perovskites with the halogen substitution of the order (Cl → Br → I). However, the predicted energy gap values are still important information toward specific applications of these systems.

Concerning the A-site cation substitution, the increased electronegativity of the N-atom compared to the S-atom and the decrease in size for substitutions of the order (TMS → FA → MA) result in a unit cell contraction and a reduction in the bandgap. The bandgap energies are affected indirectly by the A-site substitution through the substantial modification of the structural features. The FA- and MA-based systems exhibit triclinic lattices, in contrast to the cubic lattices of TMS-based systems, due to symmetry lowering. Moreover, the larger size of the TMS compared to the FA and MA cations results in a significant tilt of the octahedra that deviates from the archetype ‘defect’ perovskite structure.

The systems under investigation are wide-bandgap semiconductors, and, depending on their bandgap energy, they are suitable as UV detectors and high temperature sensors, as thermal sensors, as photocatalysts, as thin-film transistors, in the design of deep UV light-emitting diodes, in high-power electronics, in the design of UV lasers, and in LEDs. Experimental investigations to synthesize these series of compounds are currently in progress.

## Figures and Tables

**Table 1:** Calculated values for bandgap energy in eV for TMS_2_ZrCl_6_ using various functionals.

Functional	E_gap_
GGA-PBE	3.92
MBJ	5.23
HSEsol	4.69
HSE06	5.22
Experimental ^a^	5.1

^a^ Reference [20].

**Table 2:** Calculated bandgap energy, E_gap_ (eV), lattice constants of the unit cell (Å), the shortest distance between halogen–halogen in the same octahedra, d* (Å), and in nearby octahedra, d** (Å) of TMS_2_ZrX_6_.

Compound	E_gap_ (GGA-PBE)	E_gap_ (HSE06)	E_gap_ Expt	Crystal System	Lattice Constants	d*	d**
TMS_2_ZrCl_6_	3.92	5.22	5.1 ^a^	cubic	12.65	3.51	4.90
TMS_2_ZrBr_6_	3.13	4.05	-	cubic	12.86	3.73	4.78
TMS_2_ZrI_6_	2.21	2.97	-	cubic	13.49	4.06	4.87

^a^ Reference [20].

**Table 3:** Calculated bandgap energy, E_gap_ (eV), lattice constants of the unit cell (Å), the shortest distance between halogen–halogen in the same octahedra, d* (Å), and in nearby octahedra, d** (Å) of MA_2_ZrX_6_.

Compound	E_gap_ (GGA-PBE)	E_gap_ (HSE06)	Crystal System	Lattice Constants				
				a	b	c	d*	d**
MA_2_ZrCl_6_	2.88	4.06	triclinic	11.26	10.84	11.18	3.40	4.13
MA_2_ZrBr_6_	2.11	3.14	triclinic	11.64	11.23	11.59	3.63	4.21
MA_2_ZrI_6_	1.21	2.11	triclinic	12.30	11.92	12.28	3.98	4.39

**Table 4:** Calculated bandgap energy, E_gap_ (eV), lattice constants of the unit cell (Å), the shortest distance between halogen–halogen in the same octahedra, d* (Å), and in nearby octahedra, d** (Å) of FA_2_ZrX_6_.

Compound	E_gap_ (GGA-PBE)	E_gap_ (HSE06)	Crystal System	Lattice Constants			d*	d**
				a	b	c		
FA_2_ZrCl_6_	3.70	4.81	triclinic	11.94	9.94	11.49	3.47	4.18
FA_2_ZrBr_6_	2.87	3.89	triclinic	12.40	10.23	11.98	3.70	4.12
FA_2_ZrI_6_	1.86	2.54	triclinic	13.04	10.80	12.69	4.02	4.37

## Data Availability

The original contributions presented in this study are included in the article. Further inquiries can be directed to the corresponding authors.
